# Multi-pose-based convolutional neural network model for diagnosis of patients with central lumbar spinal stenosis

**DOI:** 10.1038/s41598-023-50885-9

**Published:** 2024-01-02

**Authors:** Seyeon Park, Jun-Hoe Kim, Youngbin Ahn, Chang-Hyun Lee, Young-Gon Kim, Woon Tak Yuh, Seung-Jae Hyun, Chi Heon Kim, Ki-Jeong Kim, Chun Kee Chung

**Affiliations:** 1 Transdisciplinary Department of Medicine & Advanced Technology, Seoul National University Hospital, 101 Daehak-Ro, Jongro-Gu, Seoul, 03080 Republic of Korea; 2 Department of Neurosurgery, Seoul National University Hospital, 101 Daehak-Ro, Jongro-Gu, Seoul, 03080 Republic of Korea; 3 Department of Neurosurgery, Seoul National University College of Medicine, Seoul, Republic of Korea; 4 Department of Neurosurgery, Seoul National University Bundang Hospital, Seongnam, Republic of Korea; 5 Department of Brain and Cognitive Sciences, Seoul National University College of Natural Sciences, Seoul, Republic of Korea

**Keywords:** Computer science, Musculoskeletal system, Radiography, Diagnostic markers, Image processing, Machine learning, Computational biology and bioinformatics, Anatomy, Diseases

## Abstract

Although the role of plain radiographs in diagnosing lumbar spinal stenosis (LSS) has declined in importance since the advent of magnetic resonance imaging (MRI), diagnostic ability of plain radiographs has improved dramatically when combined with deep learning. Previously, we developed a convolutional neural network (CNN) model using a radiograph for diagnosing LSS. In this study, we aimed to improve and generalize the performance of CNN models and overcome the limitation of the single-pose-based CNN (SP-CNN) model using multi-pose radiographs. Individuals with severe or no LSS, confirmed using MRI, were enrolled. Lateral radiographs of patients in three postures were collected. We developed a multi-pose-based CNN (MP-CNN) model using the encoders of the three SP-CNN model (extension, flexion, and neutral postures). We compared the validation results of the MP-CNN model using four algorithms pretrained with ImageNet. The MP-CNN model underwent additional internal and external validations to measure generalization performance. The ResNet50-based MP-CNN model achieved the largest area under the receiver operating characteristic curve (AUROC) of 91.4% (95% confidence interval [CI] 90.9–91.8%) for internal validation. The AUROC of the MP-CNN model were 91.3% (95% CI 90.7–91.9%) and 79.5% (95% CI 78.2–80.8%) for the extra-internal and external validation, respectively. The MP-CNN based heatmap offered a logical decision-making direction through optimized visualization. This model holds potential as a screening tool for LSS diagnosis, offering an explainable rationale for its prediction.

## Introduction

Since the development of magnetic resonance imaging (MRI), the importance of plain radiographs for diagnosing lumbar spinal stenosis (LSS) has been gradually declining^1^. However, MRI is still expensive, time-consuming, and not available in all medical institutions, particularly in space where portable equipment is required. In these cases, plain radiography is advantageous because of its low cost, quick scan time, immediate readability, and small/portable equipment.

Recently, there has been a growing interest in artificial intelligence (AI). One of the most popular AI algorithms is the convolutional neural network (CNN). The CNN consists of a convolution layer, pooling layer, and fully connected layer. It is especially useful for finding patterns to recognize images, learning directly from data, and finding patterns to classify images^2,3^. CNNs are also widely used in medical image classification tasks. Recent studies have shown that the diagnostic ability of plain radiographs has been much improved using AI for diagnosing lung cancer, pneumothorax, rheumatoid arthritis, moyamoya disease, and COVID-19 infection^4–8^. In addition, previous studies reported that X-ray images were used to automatically measure the Cobb angle of scoliosis and the joint space width of knee joints^9,10^. Although AI-reinforced plain radiographs are promising, only a few are practically useful and are used in clinical care. This is because the developed model may still need to reach the high accuracy required in clinical practice, or the F1-score may not be high even if the accuracy is high^4,11,12^.

The diagnosis of LSS using plain radiographs may be probable because factors that comprise of LSS, such as decreased intervertebral disc height, bony spur, facet joint degeneration, and pedicle length, can be observed on plain radiographs. However, this does not mean that plain radiographs can replace MRI in diagnosing LSS, as the role of AI is only to provide assistance. Our previous study reported a newly developed single pose-based CNN (SP-CNN) model that demonstrated 83% accuracy for diagnosing LSS^13^. Although the model demonstrated high performance, its use in the clinical setting is still limited because diagnosing LSS using only a single radiograph is a near impossible task. In the clinical setting, spine surgeons do not diagnose the disease based on a single radiograph; instead, they observed radiographs from multiple view directions and positions, e.g., anterior–posterior (AP) and lateral views during flexion, neutral, and extension. Such multi-pose radiographs assist in differentiating image variations caused by postural changes, including instability and artifacts (e.g., bowel gas, vascular calcification, or underwear band).

To overcome the limitation of the SP-CNN model, we developed a multi-pose-based CNN (MP-CNN) model using lateral radiographs in three different postures. To validate its effectiveness, we used four different algorithms commonly used in CNN methods to evaluate it in internal and external validation sets.

## Materials and methods

### Ethics statements

This study was conducted in accordance with the Declaration of Helsinki, and the study protocol was approved by the Institutional Research Board of Seoul National University Bundang Hospital (approval number: B-2106/690-108) and Seoul National University Hospital (approval number: J-2107-107-1235). The requirement for informed consent was waived by the Institutional Research Board of Seoul National University Bundang Hospital (approval number: B-2106/690-108) and Seoul National University Hospital (approval number: J-2107-107-1235) owing to the retrospective nature of this study.

### Image acquisition

This study used plain lateral radiographs of the lumbar spine of patients who met the following inclusion criteria: (1) adult patients aged ≥ 18 years, (2) patients who took an MRI following lateral radiography between May 1, 2005, and December 31, 2017, and (3) patients with “severe central canal stenosis,” “no stenosis,” or no mention of stenosis in the formal MRI reports by radiologists. The exclusion criteria were as follows: (1) cases where MRI was performed ≥ 5 years after the plain radiography, (2) patients with any foreign body, e.g., a pedicle screw, interbody cage, interspinous device, or artificial hip joint, and (3) patients who did not take radiographs on all 3 positions (neutral, flexion, and extension). Patients with “severe central canal stenosis” on formal MRI reports by radiologists were allocated in the LSS group, whereas those with “no stenosis” or with no mention of stenosis were categorized in the non-LSS group.

All images were split into a training set (80%) and a test set (20%). The training set involved five-fold cross validation, where 20% of the training set was used for model validation during the training. Therefore, 766 of the 3831 patients were included in the holdout test set (Test A). For the MP-CNN model, radiographs of all three postures were used simultaneously as inputs to the model. Thus, one case contained three radiographs, but the number of cases and participants was the same.

Furthermore, we consecutively collected internal and external validation datasets in addition to the training dataset to evaluate generalization capabilities of the models. For the extra-internal validation, 100 participants in the LSS group and 99 in the non-LSS group from the same institution were consecutively enrolled with 597 radiographs (LSS, 300; non-LSS, 297) from January 2018 to December 2020 (Test B). For external validation, 100 patients for each group were consecutively enrolled with 600 radiographs (LSS, 300; non-LSS, 300) from another hospital between January 2010 and December 2020 (Test C).

### Data preprocessing

The original radiographs were preprocessed to effectively train the deep learning model. Histogram equalization was applied for pixel normalization, followed by cropping to set the region of interest to the center of the image and resizing it to 700 × 600 pixels. Zooming, rotation, and horizontal and vertical flips were used to augment the data. Our previous study described preprocessing in detail^13^. In the present study, three different cropped images were used together as input of the MP-CNN model. The proposed MP-CNN model was composed of three different encoders for each posture so that the radiographs of different postures can be trained independently. Subsequently, concatenated feature maps were generated from these encoders. Ultimately, the features of each posture extracted from the three posture images are fused to predict LSS.

### Construction of the multi-pose model

**Figure 1:**
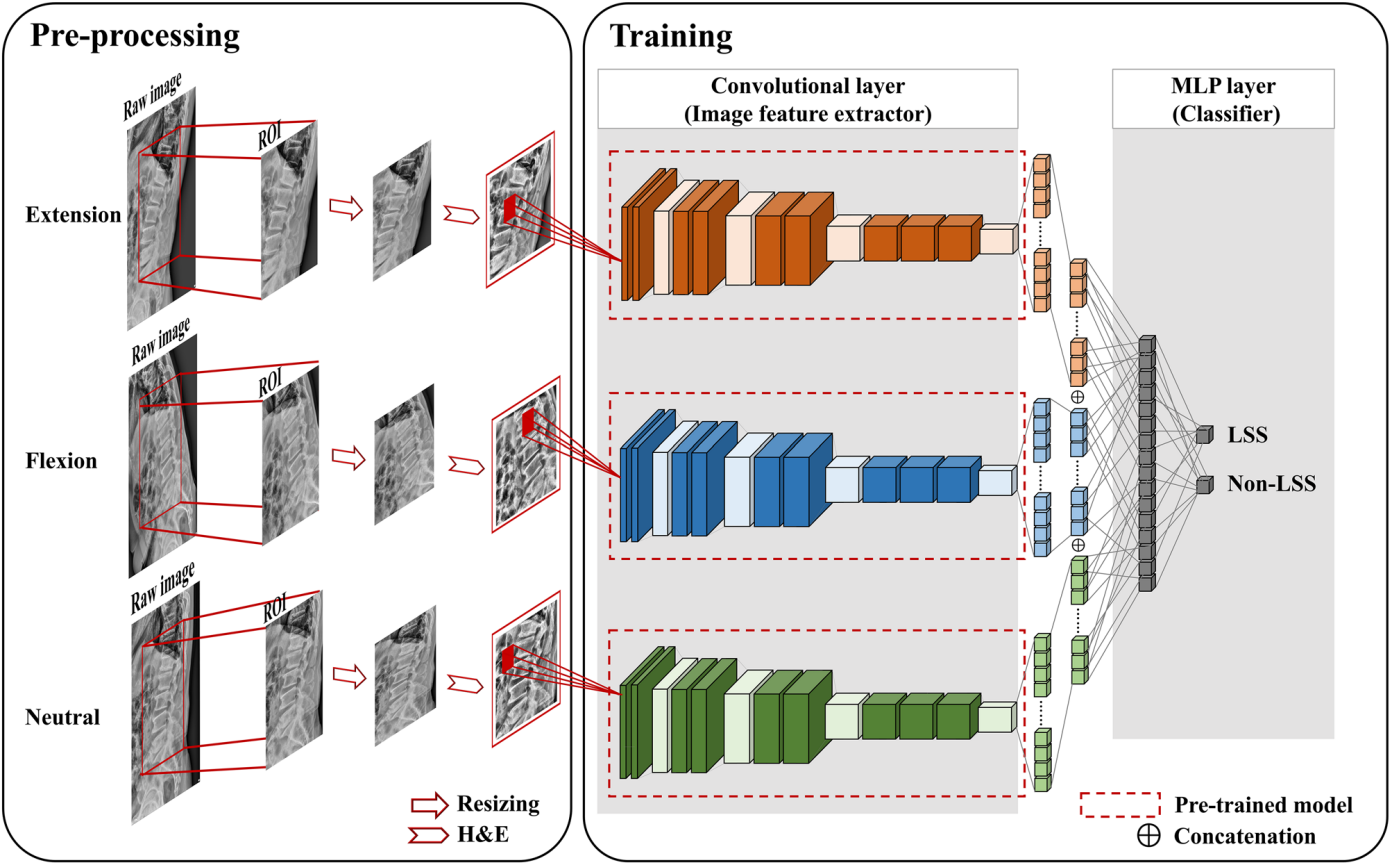
Workflow of the multi-pose model to classify severe lumbar spinal stenosis (LSS). The first part in the convolutional layer part is for the extraction of spatial features extracted by three pre-trained single-pose-based convolutional neural network models with non-trainable parameters, and the second part in multi-layer perceptron (MLP) is for classifying patients using the concatenated features. H&E Histogram equalization.

Figure 1 shows the diagram of the proposed method, which consists of two parts, a convolutional layer to extract image features using SP-CNN models for three different postures, and a multi-layer perceptron (MLP) layer to classify LSS. First, radiographs of the three different postures were simultaneously fed into each encoder. The final feature maps from each encoder were flattened and concatenated. The weights of the three different SP-CNN models were not shared and were all independent. Second, an MLP-based classifier was trained with the merged features to classify LSS. During training, the parameters of each encoder were frozen, and the parameters of the MLP layer were set to be trainable. Therefore, we validated the feasibility of MP-CNN by comparing the classification performance of models that applied the MP structure to various CNN algorithms (VGG16^14^, VGG19^14^, ResNet50^15^, and EfficientNet-B1^16^). For the hyperparameters, stochastic gradient descent was used as an optimizer; the learning rate was 0.0005, and cross-entropy was used as a loss function. The batch size was set to four, and the epoch for learning the model was set to 1000, with early stopping at an impatience of 10. Additionally, we chose the model with the lowest loss value in the validation set to select the best model. All processing, including the pre-processing and training models in this study, was performed in a shared computing facility comprising Intel Core i7-10,700 CPUs and NVIDIA RTX 2080Ti GPUs.

### Normalized heatmap

Gradient-weighted class activation mapping (Grad-CAM) is a method used to obtain a heatmap to localize the part of the image that has the greatest influence on the decision in the CNN models with pixel values ranging from 0 to 255^17^. By applying Grad-CAM to the MP-CNN model, visualization maps for each posture were generated. Owing to substantial variations in feature values among the three postures encoders (neutral, flexion, and extension) of the MP-CNN model, we initially normalized the feature values within each posture encoder of the MP-CNN model before considering normalization across all three postures. Normalization was based on the maximum value of all features of three postures. After feature normalization, we calculated the probability of LSS. This enabled us to generate a visualization map indicating the influential posture images among the patient's three postures and the regions with the most considerable impact on LSS prediction.

### Evaluation metrics and statistical analysis

Our comparison centered on the Area Under the Receiver Operating Characteristic (AUROC), as it is a robust performance measure in binary classification tasks that is not dependent on specific probability thresholds^18,19^. We compared the performance of MP-CNN models with SP-CNN models for the most commonly used CNN-based algorithms of VGG16, VGG19, ResNet50, and EfficientNet-B1 in terms of AUROC^20^. Additionally, accuracy, sensitivity, specificity, positive predictive value (PPV), and negative predictive value (NPV) were measured with a threshold of 0.5 (Model output 0–1). To determine any statistically significant differences between the AUROC of each SP-CNN model and the MP-CNN model, statistical analysis was performed using a paired t-test with a p-value < 0.05 defined as statistically significant (two-sided).

## Results

**Figure 2:**
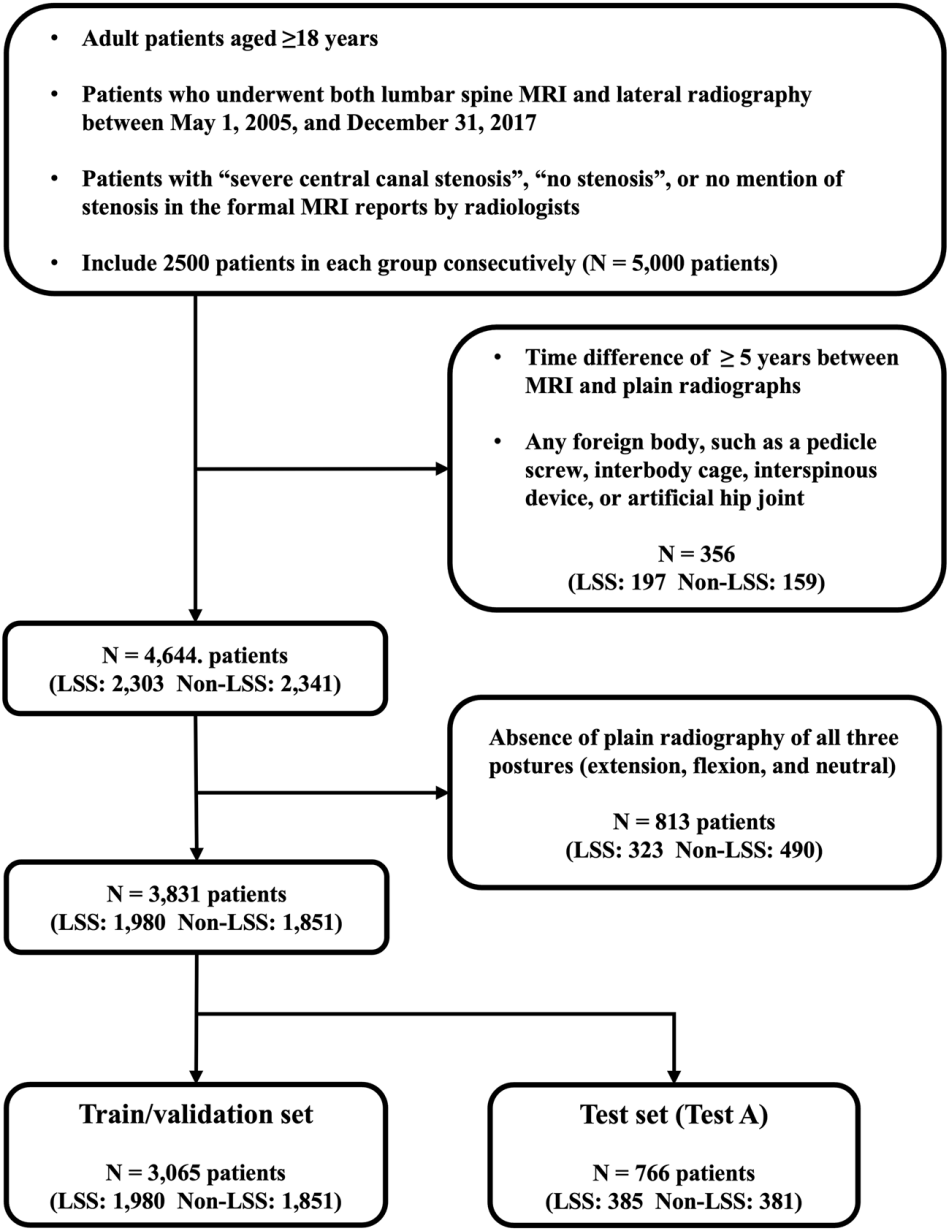
Flow diagram of included patients.

Participant enrollment is detailed in Fig. 2. We consecutively identified 2500 patients in each group. After applying the exclusion criteria, the dataset comprised 2303 patients with LSS and 2341 individuals without LSS. Among the total cohort, only those with radiographs of all three postures were selected, which included 1,980 participants with LSS and 1851 participants without LSS. Therefore, the total number of radiographs used in this study were 5940 and 5553 in the LSS and non-LSS groups, respectively. In the original dataset, the mean age (± standard deviation) was 69.2 ± 11.2 years in the LSS group and 49.2 ± 15.8 years in the non-LSS group, which was statistically significantly different (*p* < 0.001). The extra-internal validation (Test B) also revealed a significant age difference between the two groups (*p* < 0.001). In the external validation (Test C), no substantial difference was detected in the mean age of the two groups (LSS, 69.1 ± 13.1 years; non-LSS, 69.0 ± 8.6 years;* p* = 0.886). The female patient ratios for the LSS/non-LSS group were 60.3%/42.4% in the original dataset, 62.0%/54.5% in Test B, and 58.0%/60.0% in Test C. No significant difference in sex was observed between the LSS and non-LSS groups in any dataset (*p* = 0.08 [original dataset], *p* = 0.16 [training dataset], *p* = 0.97 [Test A], *p* = 0.06 [Test B], *p* = 0.06 [Test C]).

### Comparison of four pre-trained algorithms by ImageNet and posture model

We used four algorithms pre-trained on ImageNet. We compared the average performance of the MP-CNN model extracted using each algorithm with the average performance of the three SP-CNN models, as shown in Supplementary Table 1. The results showed how much the MP-CNN model improved over the three posture SP-CNN models. In Test A, the MP-CNN model showed improvements of 1.8%, 2.5%, and 2.1% over the three SP-CNN models (neutral, extension, and flexion postures), respectively. In Test B, the MP-CNN model showed improvements of 2.0%, 2.0%, and 2.4% over the three SP-CNN models, respectively. Finally, in Test C, the MP-CNN model showed improvements of 0.7%, 2.6%, and 5.0% over the three SP-CNN models, respectively.

**Figure 3:**
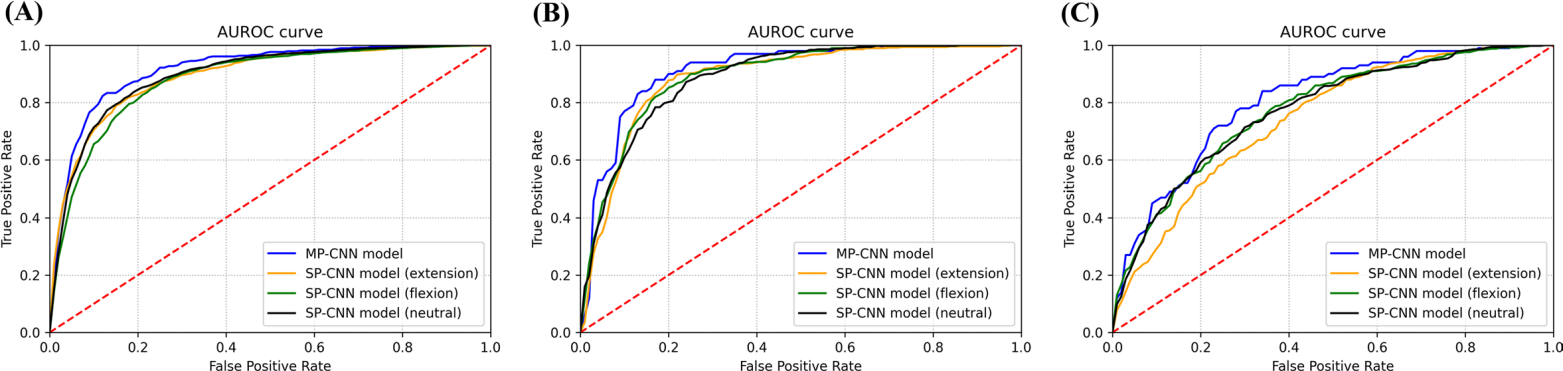
Comparison of the area under the receiver operating characteristic (AUROC) curves of ResNet50-based multi-pose-based convolutional neural network (MP-CNN) model and three different single-pose-based convolutional neural network (SP-CNN) models for different test sets, (**A**) Test A, (**B**) Test B, and (**C**) Test C.

In the internal validation (Test A), the VGG19-based MP-CNN model showed the highest AUROC of 92.0% (95% confidence interval [CI], 91.6–92.3%), followed by 91.4% (95% CI, 90.9–91.8%) in the ResNet50-based MP-CNN, and 91.4% (95% CI, 91.0–91.7%) in the VGG16-based MP-CNN, as shown in Table 1. The other performance metrics demonstrated similar trends for all the algorithms and models. In the extra-internal validation (Test B), the MP-CNN models exhibited a larger AUROC than the SP-CNN models for all four algorithms, as shown in Table 2. The largest AUROC in Test B was 91.4% (95% CI 90.3–92.5%) in the VGG19-based MP-CNN model, followed by 91.3% (95% CI 90.7–91.9%) in the ResNet50-based MP-CNN model, and 91.0% (95% CI 90.5–91.5%) in the VGG16-based MP-CNN model. The EfficientNet-B1-based MP-CNN model exhibited a lower performance than the other models. The overall performance of the external validation test (Test C) was inferior to those of Tests A and B, as shown in Table 3. The best model was the ResNet50-based MP-CNN model, which showed an AUROC of 79.5% (95% CI 78.2–80.8%), followed by the VGG-based SP-CNN model for neutral posture with an AUROC of 77.6% (95% CI 75.4–79.8%). The receiver operating characteristic curves of the MP-CNN and the three SP-CNN models based on the ResNet50 architecture are shown in Fig. 3. In terms of AUROC, the ResNet50-based MP-CNN model outperformed the other ResNet50-based SP-CNNs in all three test sets; however, the model with the smallest AUROC differed between the tests. The model with the smallest AUROCs were the SP-CNN for flexion in Test A, SP-CNN for the neutral posture in Test B, and SP-CNN for extension in Test C.

**Table 1:** Performance metrics with Test A for MP-CNN model vs SP-CNN model.

Algorithm	Model	AUROC (95% CI)	Accuracy (95% CI)	Sensitivity (95% CI)	Specificity (95% CI)	PPV (95% CI)	NPV (95% CI)
ResNet50	MP-CNN	**0.914 (0.909–0.918)**	0.842 (0.837–0.846)	0.850 (0.817–0.883)	0.833 (0.794–0.872)	0.839 (0.812–0.866)	0.848 (0.826–0.860)
	SP-CNN(Neu)	0.896* (0.890–0.902)	0.819 (0.812–0.827)	0.850 (0.828–0.872)	0.788 (0.758–0.818)	0.803 (0.784–0.822)	0.839 (0.824–0.854)
	SP-CNN(Flx)	0.882* (0.875–0.888)	0.809 (0.796–0.823)	0.834 (0.811–0.857)	0.785 (0.773–0.797)	0.797 (0.786–0.807)	0.824 (0.804–0.845)
	SP-CNN(Ext)	0.893* (0.890–0.897)	0.813 (0.800–0.825)	0.837 (0.808–0.867)	0.788 (0.756–0.820)	0.801 (0.779–0.822)	0.829 (0.807–0.850)
VGG19	MP-CNN	**0.920 (0.916–0.923)**	0.854 (0.845–0.863)	0.855 (0.836–0.873)	0.854 (0.837–0.872)	0.856 (0.842–0.871)	0.852 (0.844–0.860)
	SP-CNN(Neu)	0.900* (0.894–0.906)	0.828 (0.821–0.836)	0.846 (0.815–0.878)	0.810 (0.787–0.833)	0.818 (0.805–0.831)	0.845 (0.828–0.862)
	SP-CNN(Flx)	0.901* (0.898–0.904)	0.836 (0.833–0.839)	0.846 (0.840–0.851)	0.826 (0.818–0.834)	0.831 (0.825–0.837)	0.822 (0.809–0.835)
	SP-CNN(Ext)	0.898* (0.891–0.905)	0.827 (0.820–0.834)	0.842 (0.827–0.857)	0.812 (0.797–0.827)	0.819 (0.809–0.830)	0.832 (0.814–0.850)
VGG16	MP-CNN	**0.914 (0.910–0.917)**	0.850 (0.843–0.857)	0.855 (0.846–0.863)	0.846 (0.834–0.857)	0.848 (0.839–0.858)	0.848 (0.826–0.860)
	SP-CNN(Neu)	0.896* (0.891–0.901)	0.824 (0.816–0.832)	0.856 (0.828–0.877)	0.792 (0.779–0.805)	0.806 (0.798–0.814))	0.839 (0.824–0.854)
	SP-CNN(Flx)	0.888* (0.882–0.894)	0.819 (0.814–0.824)	0.825 (0.808–0.843)	0.812 (0.805–0.819)	0.816 (0.813–0.819)	0.824 (0.804–0.845)
	SP-CNN(Ext)	0.895* (0.889–0.901)	0.819 (0.813–0.825)	0.839 (0.816–0.863)	0.798 (0.786–0.811)	0.808 (0.803–0.814)	0.829 (0.807–0.850)
EfficientNet-B1	MP-CNN	**0.905 (0.901–0.908)**	0.839 (0.833–0.846)	0.840 (0.825–0.855)	0.839 (0.829–0.849)	0.841 (0.833 –0.848)	0.839 (0.827–0.851)
	SP-CNN(Neu)	0.887* (0.875–0.898)	0.815 (0.804–0.825)	0.823 (0.802–0.843)	0.806 (0.789–0.824)	0.811 (0.799–0.824)	0.819 (0.803–0.835)
	SP-CNN(Flx)	0.881* (0.870–0.891)	0.817 (0.803–0.832)	0.825 (0.811–0.839)	0.810 (0.793–0.827)	0.814 (0.799–0.830)	0.821 (0.807–0.835)
	SP-CNN(Ext)	0.883* (0.877–0.889)	0.811 (0.804–0.818)	0.819 (0.795–0.842)	0.804 (0.790–0.819)	0.809 (0.801–0.817)	0.815 (0.798–0.833)

The best values are in bold.

Statistical analysis was performed using a paired t-test to compare the AUROC of each SP-CNN model with that of the MP-CNN model. An asterisk (*) means statistically significant difference (*p* < 0.05).

*AUROC* area under the receiver operating characteristics curve, *PPV* positive predictive value, *NPV* negative predictive value, *CI* confidence interval, *MP-CNN* multi pose-based convolutional neural network model, *SP-CNN* single pose-based convolutional neural network, *Neu* neutralposture, *Flx* flexion posture, *Ext* extension posture.

**Table 2:** Performance metrics with Test B for MP-CNN model vs SP-CNN model.

Algorithm	Model	AUROC (95% CI)	Accuracy (95% CI)	Sensitivity (95% CI)	Specificity (95% CI)	PPV (95% CI)	NPV (95% CI)
ResNet50	MP-CNN	**0.913 (0.907–0.919)**	0.830 (0.805–0.855)	0.784 (0.729–0.839)	0.877 (0.854–0.900)	0.866 (0.845–0.887)	0.803 (0.764–0.842)
	SP-CNN(Neu)	0.890* (0.882–0.898)	0.805 (0.789–0.821)	0.800 (0.778–0.822)	0.810 (0.780–0.840)	0.810 (0.787–0.834)	0.801 (0.783–0.818)
	SP-CNN(Flx)	0.896* (0.887–0.905)	0.817 (0.810–0.824)	0.780 (0.768–0.792)	0.855 (0.840–0.869)	0.844 (0.832–0.857)	0.794 (0.785–0.802)
	SP-CNN(Ext)	0.890* (0.880–0.901)	0.821 (0.801–0.841)	0.800 (0.814–0.871)	0.842 (0.814–0.871)	0.838 (0.817–0.859)	0.809 (0.773–0.845)
VGG19	MP-CNN	**0.914 (0.903–0.925)**	0.829 (0.810–0.848)	0.782 (0.758–0.806)	0.877 (0.852–0.901)	0.865 (0.842–0.889)	0.802 (0.790–0.813)
	SP-CNN(Neu)	0.893* (0.877–0.909)	0.818 (0.789–0.847)	0.778 (0.717–0.839)	0.859 (0.779–0.939)	0.849 (0.783–0.915)	0.804 (0.789–0.818)
	SP-CNN(Flx)	0.895* (0.882–0.908)	0.819 (0.798–0.840)	0.782 (0.746–0.818)	0.857 (0.841–0.872)	0.846 (0.829–0.863)	0.790 (0.767–0.813)
	SP-CNN(Ext)	0.890* (0.875–0.905)	0.815 (0.804–0.826)	0.774 (0.757–0.791)	0.857 (0.826–0.887)	0.846 (0.821–0.872)	0.813 (0.798–0.838)
VGG16	MP-CNN	**0.910 (0.905–0.915)**	0.835 (0.829–0.842)	0.782 (0.764–0.800)	0.889 (0.875–0.903)	0.877 (0.865–0.889)	0.803 (0.764–0.842)
	SP-CNN(Neu)	0.889* (0.882–0.895)	0.819 (0.806–0.832)	0.796 (0.779–0.813)	0.842 (0.829–0.856)	0.836 (0.823–0.849)	0.801 (0.783–0.818)
	SP-CNN(Flx)	0.889* (0.884–0.895)	0.819 (0.804–0.834)	0.770 (0.737–0.803)	0.869 (0.849–0.888)	0.856 (0.839– 0.873)	0.794 (0.785–0.802)
	SP-CNN(Ext)	0.892* (0.885–0.898)	0.824 (0.808–0.841)	0.808 (0.777–0.839)	0.840 (0.833–0.848)	0.836 (0.827–0.845)	0.809 (0.773–0.845)
EfficientNet-B1	MP-CNN	**0.899 (0.894–0.905)**	0.800 (0.792–0.808)	0.714 (0.693–0.735)	0.887 (0.879–0.894)	0.865 (0.859– 0.870)	0.755 (0.742–0.767)
	SP-CNN(Neu)	0.883* (0.873–0.893)	0.800 (0.780–0.820)	0.734 (0.701–0.767)	0.867 (0.850–0.884)	0.848 (0.830–0.866)	0.764 (0.741–0.787)
	SP-CNN(Flx)	0.877 (0.857–0.898)	0.798 (0.778–0.818)	0.726 (0.697–0.755)	0.871 (0.856–0.885)	0.850 (0.832–0.868)	0.759 (0.738–0.780)
	SP-CNN(Ext)	0.866* (0.848–0.884)	0.790 (0.778–0.802)	0.735 (0.714–0.755)	0.846 (0.826–0.867)	0.829 (0.812–0.847)	0.759 (0.746–0.773)

The best values are in bold.

Statistical analysis was performed using a paired t-test to compare the AUROC of each SP-CNN model with that of the MP-CNN model. An asterisk (*) means statistically significant difference (*p* < 0.05).

*AUROC* area under the receiver operating characteristics curve, *PPV* positive predictive value, *NPV* negative predictive value, *CI* confidence interval, *MP-CNN* multi pose-based convolutional neural network model, *SP-CNN* single pose-based convolutional neural network, *Neu* neutralposture, *Flx* flexion posture, *Ext* extension posture.

**Table 3:** Performance metrics with Test C for MP-CNN model vs SP-CNN model.

Algorithm	Model	AUROC (95% CI)	Accuracy (95% CI)	Sensitivity (95% CI)	Specificity (95% CI)	PPV (95% CI)	NPV (95% CI)
ResNet50	MP-CNN	**0.795 (0.782–0.808)**	0.667 (0.631–0.703)	0.930 (0.902–0.958)	0.404 (0.308–0.500)	0.612 (0.578–0.646)	0.856 (0.829–0.883)
	SP-CNN(Neu)	0.769 (0.748–0.790)	0.642 (0.614–0.670)	0.922 (0.897–0.947)	0.362 (0.284–0.440)	0.593 (0.569–0.616)	0.826 (0.800–0.852)
	SP-CNN(Flx)	0.773 (0.747–0.798)	0.651 (0.635–0.667)	0.900 (0.867–0.933)	0.402 (0.340–0.464)	0.602 (0.585–0.619)	0.806 (0.778–0.883)
	SP-CNN(Ext)	0.742* (0.726–0.758)	0.661 (0.638–0.684)	0.886 (0.848–0.924)	0.436 (0.366–0.506)	0.612 (0.591– 0.634)	0.798 (0.760–0.836)
VGG19	MP-CNN	0.772 (0.743–0.801)	0.683 (0.657–0.709)	0.920 (0.879–0.961)	0.446 (0.377–0.515)	0.625 (0.602–0.649)	0.857 (0.800–0.914)
	SP-CNN(Neu)	0.776* (0.754–0.798)	0.667 (0.642–0.692)	0.926 (0.883–0.969)	0.408 (0.329–0.486)	0.612 (0.589–0.648)	0.883 (0.838–0.928)
	SP-CNN(Flx)	0.743 (0.707–0.780)	0.648 (0.624–0.672)	0.920 (0.872–0.968)	0.376 (0.315–0.437)	0.596 (0.579–0.635)	0.859 (0.808–0.911)
	SP-CNN(Ext)	0.721* (0.695–0.747)	0.665 (0.635–0.695)	0.858 (0.794–0.922)	0.472 (0.395–0.549)	0.620 (0.595–0.645)	0.780 (0.714–0.847)
VGG16	MP-CNN	**0.775 (0.743–0.807)**	0.667 (0.636–0.698)	0.952 (0.935–0.969)	0.382 (0.313–0.451)	0.608 (0.582–0.633)	0.890 (0.856–0.924)
	SP-CNN(Neu)	0.769 (0.727–0.811)	0.630 (0.600–0.660)	0.984 (0.971–0.997)	0.276 (0.207–0.345)	0.577 (0.556–0.599)	0.951 (0.917–0.984)
	SP-CNN(Flx)	0.743 (0.695–0.790)	0.641 (0.606–0.675)	0.903 (0.883–0.923)	0.384 (0.322–0.446)	0.590 (0.566–0.615)	0.796 (0.735–0.857)
	SP-CNN(Ext)	0.725 (0.689–0.761)	0.670 (0.652–0.688)	0.902 (0.876–0.928)	0.438 (0.394–0.482)	0.617 (0.601–0.632)	0.820 (0.784–0.856)
EfficientNet-B1	MP-CNN	**0.748 (0.727–0.768)**	0.634 (0.624–0.644)	0.944 (0.934–0.954)	0.324 (0.306–0.342)	0.583 (0.576– 0.590)	0.853 (0.830–0.876)
	SP-CNN(Neu)	0.726 (0.698–0.754)	0.604 (0.581–0.627)	0.920 (0.896–0.944)	0.288 (0.242–0.334)	0.564 (0.548–0.580)	0.783 (0.733–0.834)
	SP-CNN(Flx)	0.726* (0.701–0.751)	0.631 (0.603–0.659)	0.946 (0.938–0.954)	0.316 (0.253–0.379)	0.581 (0.561–0.601)	0.852 (0.840–0.864)
	SP-CNN(Ext)	0.700 (0.669–0.732)	0.623 (0.597–0.649)	0.888 (0.858–0.918)	0.358 (0.293–0.423)	0.581 (0.562–0.601)	0.762 (0.728–0.796)

The best values are in bold.

Statistical analysis was performed using a paired t-test to compare the AUROC of each SP-CNN model with that of the MP-CNN model. An asterisk (*) means statistically significant difference (*p* < 0.05).

*AUROC* area under the receiver operating characteristics curve, *PPV* positive predictive value, *NPV* negative predictive value, *CI* confidence interval, *MP-CNN* multi pose-based convolutional neural network model, *SP-CNN* single pose-based convolutional neural network, *Neu* neutralposture, *Flx* flexion posture, *Ext* extension posture.

### Representative cases

**Figure 4:**
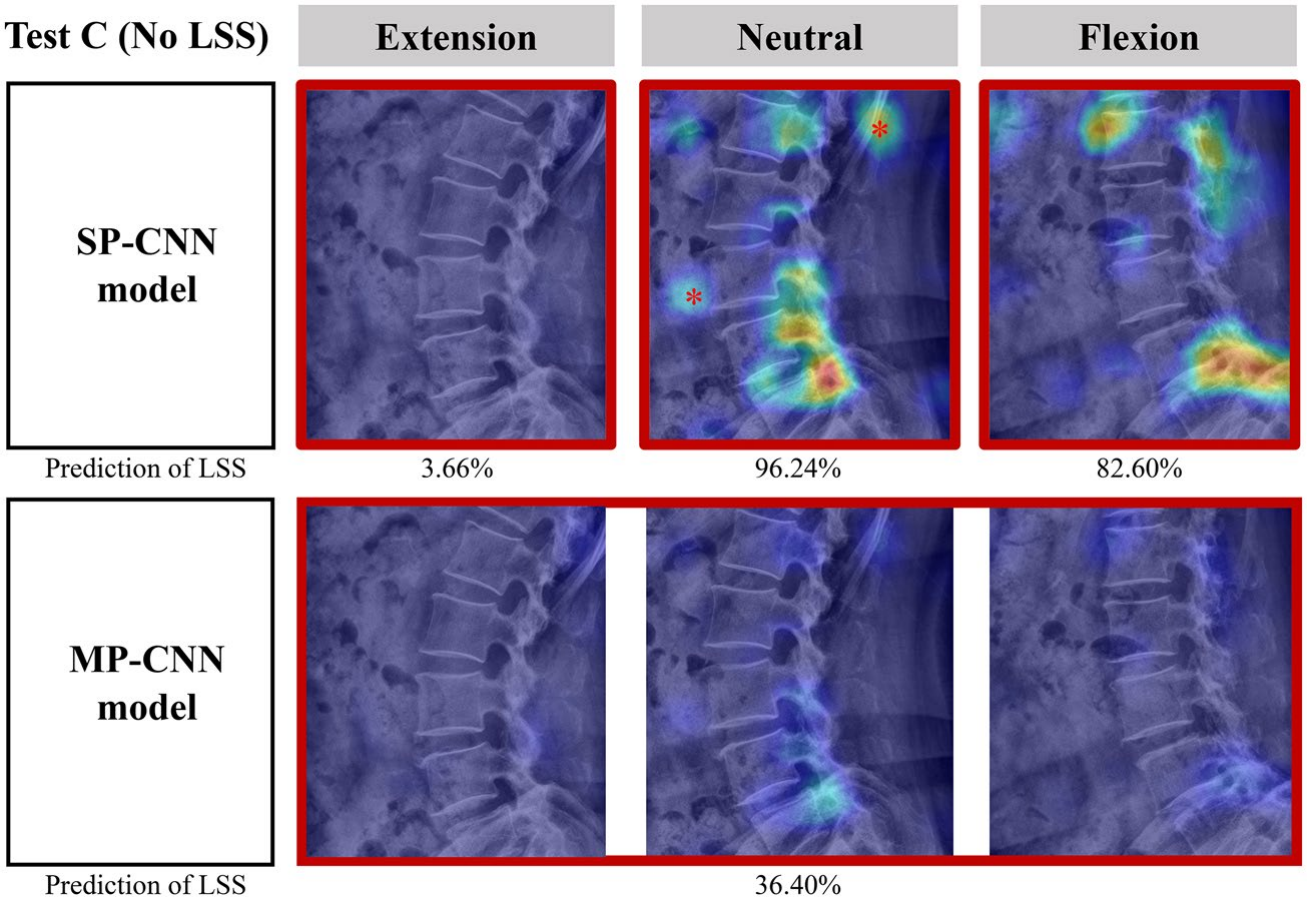
An example of heatmaps for an illustrative case that demonstrates a false positive in single-pose-based convolutional neural network (SP-CNN) models for a 78-year-old female patient without lumbar spinal stenosis (LSS). The 2 SP-CNN models (flexion and neutral posture) predicted the radiograph as LSS with probability on various spine levels and other irrelevant regions (red asterisks), but the multi-pose-based convolutional neural network (MP-CNN) model correctly predicted the radiographs as no LSS. This misprediction may have been caused by the high iliac crest line observed on both neutral and flexion images, which was comparable to that seen on extension images. MP-CNN model combines three postures and attenuates false positive lesions.

**Figure 5:**
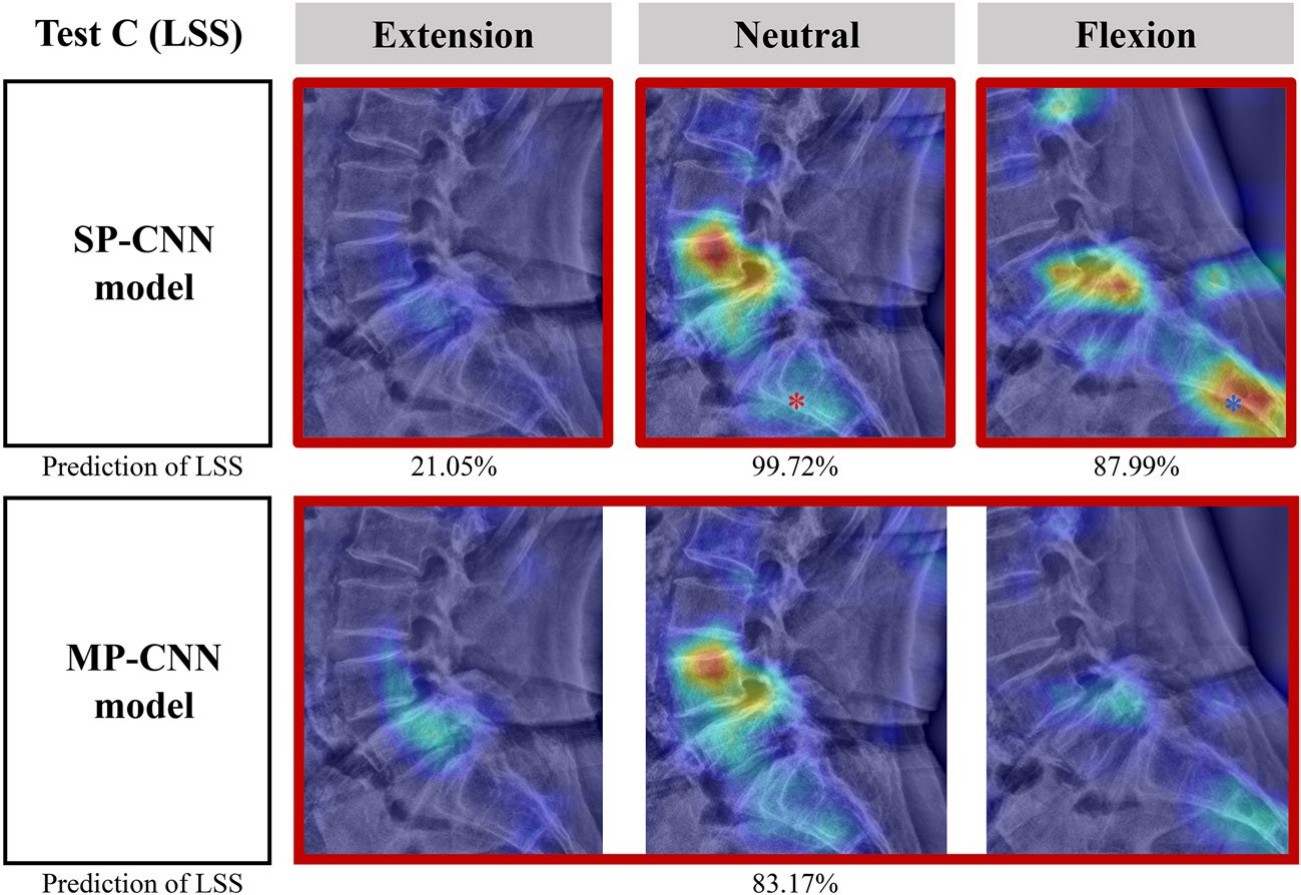
An example of heatmaps for an illustrative case that demonstrates extraction errors in single-pose-based convolutional neural network (SP-CNN) models for a 71-year-old female patient with lumbar spinal stenosis (LSS). The SP-CNN models in both neutral and flexion postures predicted LSS. However, the SP-CNN model in the neutral postures localized a probability of LSS on the sacrum (red asterisk) in addition to the foramen of L4/5. Additionally, the SP-CNN model in the flexion postures localized an even higher probability of LSS on the sacrum (blue asterisk) than on the foramen of L4/5. On the other hand, the multi-pose-based convolutional neural network (MP-CNN) model attenuated false positive and false negative results, such as iliac crest, bowel gas, spondylolisthesis, and disk height changes by postures, and accurately predicted LSS on L4/5.

Two illustrative cases are shown in Figs. 4 and 5. In the case of a 78-year-old woman without LSS, two SP-CNN models (flexion and neutral posture) predicted that her radiographs showed LSS (Fig. 4). This misprediction may have occurred because of the high iliac crest line on both neutral and flexion images. The iliac crest overlapped superior endplate and pedicle of L5 may have appeared to be LSS. In contrast, the MP-CNN model combined the three postures and attenuated false-positive lesions of the SP-CNN models. In another case of a 71-year-old female patient with LSS, the SP-CNN model for the neutral posture predicted LSS on the basis of a high probability of a foramen at the L4/5 level and a low probability of an S1 body (Fig. 5). In the SP-CNN model for the neutral posture, a warm lesion of an S1 body may be a feature-extraction error. In the SP-CNN for the flexion posture, the model predicted the patient as having LSS on basis of the high probability of an S2 body and a foramen at the L4/5 level. The SP-CNN in the extended posture falsely predicted LSS on the basis of false negatives. The MP-CNN model attenuated both false positives and false negatives, such as the iliac crest, bowel gas, spondylolisthesis, and disk height change by posture and accurately predicted LSS at the L4/5 level.

## Discussion

In terms of the AUROC, the MP-CNN model outperformed the three SP-CNN models in the performance evaluation. Of the four algorithms pretrained with ImageNet, the ResNet50-based MP-CNN model demonstrated the largest AUROCs at 79.5% in external validation, while VGG19 achieved the largest AUROC of 91.4% in extra-internal validation. This suggests that the ResNet50-based MP-CNN model may exhibit the most generalized performance. The advantage of the MP-CNN model was the attenuation of false-positive and false-negative findings.

### Model performance comparison by algorithm

We trained and validated the MP-CNN models using algorithms commonly used in the binary classification of medical images^21^. The outstanding performance of ResNet50 is likely owing to its similarity to DenseNet^22^, the most optimized algorithm for heatmap visualization^23^. Given its excellent performance, we selected the MP-CNN model with ResNet50 to validate our visual analysis. For visual analysis verification, the heatmap was optimized by normalizing the feature maps extracted from the MP-CNN models using the ResNet50 Algorithm. We concluded that the generalization performance of the ResNet50-based MP-CNN model was excellent because the structure was efficiently learned by reducing the number of parameters, even though it was a deep-layer model that used the bottleneck structure. In comparison, VGG16 and VGG19, which are structures made by simply stacking convolution blocks deeply, showed poor generalization performance because of the overfitting of the learning data as the model deepened. Moreover, EfficientNet-B1 performed the least well on all test sets compared with the MP-CNN models with the other algorithms. The number of parameters for ResNet50, VGG19, VGG16, and EfficientNet-B1 were 74.4 M, 425.8 M, 409.8 M, and 22.2 M, respectively. Because predicting LSS requires a large amount of information, we judged that EfficientNet-B1, with the fewest parameters, did not learn properly and thus showed a low performance. Additionally, because we prioritized the validation of the MP-CNN model for each algorithm, we did not experiment with hyperparameter tuning of each algorithm. Therefore, the EfficientNet-B1-based MP-CNN model may have experienced poor performance because it did not use optimized hyperparameters.

### Comparison of the MP- and SP-CNN models

Many attempts have been made to develop predictive algorithms using plain radiographs to quickly screen for diagnosing a target disease. However, these algorithms sometimes lack explanatory ability despite their high performance because of the binary nature of disease prediction algorithms^11,12^. To overcome this problem, methods that simultaneously use multiple images have been actively applied for a long time^24–26^. Rubin et al. trained a dual CNN model using chest radiographs taken in the AP and lateral views and developed a model to read chest radiographs automatically^25^. The authors reported that the performance of the models using multiple inputs was significantly superior to that of models using a single input. Other investigators have reported that CNN using multi-pose images could enhance the performance of facial recognition^27^.

### Advantages of the newly developed model

The previous SP-CNN model^13^ for diagnosing LSS was trained using radiographs of three different postures without distinction by posture, and the heatmap obtained through Grad-CAM showed that clues for diagnosing LSS were obtained in various image parts according to each posture. Although the model showed a high AUROC, predictions based on a single radiograph frequently resulted in feature-extraction errors.

The MP-CNN model appears to have advantages over the SP-CNN models. One advantage is that the MP-CNN model was designed to classify LSS by considering various clues from each posture and combining them for prediction. If clues for LSS exist in different postures, the MP-CNN model assigns more weight to this information. Conversely, if clues only exist in a single posture, the model will decrease the weight of this information when predicting LSS. This combination of information in the MLP layers is one of the reasons why the MP-CNN model performs better than the SP-CNN models. Another advantage is that the prediction errors due to artifacts (e.g., bowel gas, vascular calcification, or underwear band) and improperly taken radiographs (non-true lateral images because of scoliosis or centrally located sacrum) are decreased. Considering the error rate of the SP-CNN models caused by artifacts and improper images, the MP-CNN model concatenate extracted features from different perspectives and the MLP layers of the model can be trained better, which can lead to substantially enhanced model performance.

### Which one is the best algorithm and model

Summarizing the results of Tests A, B, and C, the VGG19-based MP-CNN model had the highest AUROCs in Tests A and B, whereas the ResNet50-based MP-CNN model had the largest AUROC in Test C among algorithms pretrained with ImageNet. Because Test C was external validation, the MP-CNN model based on ResNet50 had the best generalization performance. However, the AUROC of the ResNet50-based MP-CNN model in Test C was 79.5%, significantly lower than that observed in Tests A and B. For datasets A and B, there were many false positives for older patients in the non-LSS group and many false negatives for younger patients in the LSS group. Dataset C exhibited a mean age of the non-LSS group approximately 20 years higher than that of the other datasets, resulting in a higher frequency of false positive. Nevertheless, all negative predictive values (NPVs) in the MP-CNN model for the four algorithms were 85.0%, and the sensitivity was ≥ 90.0%. Although the performance of the MP-CNN model was poor in older patients in the non-LSS group in Test C, its performance was good in younger patients in the non-LSS group. This could be caused by the sharp image characteristics of Test C compared with those of Tests A and B, which caused the output scale values in Test C to be mismatched.

### Limitations and future directions

Our study had several limitations. First, plain radiographs have clear limitations in diagnosing LSS. Radiographs suggest only a rough distinction and are never a substitute for MRI. Because of the limitation of radiographs, CNN models using radiographic data also have limited accuracy. The CNN model only suggests suspected lesion with color mapping on radiographs to aid physicians’ decisions. It may help doctors to obtain more information from radiographs. Second, our AI model used only “severe” and “non-stenosis” patients; thus, patients with mild to moderate stenosis may have been missed. The reason for our comparison between “non-stenosis” and “severe stenosis” was to ensure clinical usefulness and a clear distinction. When training AI, it is necessary to define absolutes, true and false. Even when doctors make diagnoses based on MRI findings, there is a lack of perfect consistency in distinguishing “moderate stenosis” from “mild” stenosis. Strictly speaking, it may be impossible because spinal stenosis represents a continuous value rather than a categorical one. However, the distinction between “severe stenosis” and “non-stenosis” leaves no margin for ambiguity. In the clinical setting, distinguishing patients with severe stenosis who may require MRI evaluations and further treatment may be more important and effective than diagnosing severity. Third, the model performance was lower in Test C than in Tests A and B. A probable reason for this is that the model indicates potential overfitting of X-ray images to the visual context displaying age-related factors. Consequently, it is plausible to assume that the distinct age distributions between the training dataset and Test C considerably impacted the suboptimal performance. To address this issue, including images with heterogeneous features in the training data may help compensate for the performance differences based on the output scale. Fourth, the predictions of the MP-CNN model were correct, but the lesions on which the predictions were based differed in three postures. Among them, some cases showed LSS at multiple levels; additionally, some lesions were severe, whereas other lesions were moderate. Clinicians who use the CNN model need to consider these limitations when interpreting heatmap images. Finally, the performance of the current MP-CNN model was 80%, which is insufficient for clinical use. Moreover, owing to the insufficient number of training datasets, the deep version of the model performed worse than the light version. However, this can be improved by adding more data from multiple institutes in future studies. Furthermore, developing the current MP-CNN model using the attention mechanism^28^, which reflects input values from all or specific regions and indicates the parts that should be focused on, would enable improved performance. In addition, adding region of interest segmentation involved in LSS diagnostics to raw image preprocessing has the potential to improve model performance.

## Conclusion

The deep learning-based MP-CNN model for predicting LSS using the three postures on lumbar radiographs showed high diagnostic accuracy through internal and external validations. This model holds promise as a screening tool for LSS diagnosis, offering an explainable rationale for its prediction.

### Supplementary Information

Supplementary Information.

## Data Availability

The datasets generated during the current study are available from the corresponding author on reasonable request.
