# Supplementary material for: Multi-pose-based convolutional neural network model for diagnosis of patients with central lumbar spinal stenosis
Source: Sci Rep. 2024 Jan 2;14:203. doi: 10.1038/s41598-023-50885-9 (PMC10761871; doi:10.1038/s41598-023-50885-9)
Supplement: Supplementary file 1 — Supplementary Information. [file 41598_2023_50885_MOESM1_ESM.docx]

Supplementary Table 1 Mean performance of four pretrained algorithms by ImageNet on Test A, B, and C

| Testset | Model | AUROC  (95% CI) | Accuracy  (95% CI) | Sensitivity  (95% CI) | Specificity  (95% CI) | PPV  (95% CI) | NPV  (95% CI) |
| --- | --- | --- | --- | --- | --- | --- | --- |
| Test A | **MP-CNN** | **0.913**  **(0.910 – 0.916)** | 0.846  (0.842 – 0.851) | 0.850  (0.840 – 0.860) | 0.843  (0.832 – 0.854) | 0.846  (0.838 – 0.854) | 0.848  (0.840 – 0.855) |
|  | SP-CNN(Neu) | 0.895  (0.891 – 0.899) | 0.821  (0.817 – 0.826) | 0.844  (0.833 – 0.854) | 0.799  (0.789 – 0.809) | 0.810  (0.803 – 0.816) | 0.836  (0.828 – 0.844) |
|  | SP-CNN(Flx) | 0.888  (0.883 – 0.893) | 0.820  (0.814 – 0.827) | 0.832  (0.824 – 0.841) | 0.808  (0.800 – 0.817) | 0.815  (0.807 – 0.822) | 0.827  (0.820 – 0.835) |
|  | SP-CNN(Ext) | 0.892  (0.889 – 0.896) | 0.818  (0.813 – 0.822) | 0.834  (0.823 – 0.846) | 0.801  (0.791 – 0.811) | 0.809  (0.803– 0.816) | 0.828  (0.819 – 0.837) |
| Test B | **MP-CNN** | **0.909**  **(0.905 – 0.913)** | 0.824  (0.814 – 0.833) | 0.766  (0.745 – 0.786) | 0.882  (0.873 – 0.891) | 0.868  (0.860 – 0.876) | 0.790  (0.776 – 0.804) |
|  | SP-CNN(Neu) | 0.889  (0.885 – 0.893) | 0.811  (0.802 – 0.819) | 0.777  (0.760 – 0.794) | 0.844  (0.829 – 0.859) | 0.836  (0.824 – 0.848) | 0.790  (0.780 – 0.801) |
|  | SP-CNN(Flx) | 0.889  (0.883 – 0.896) | 0.813  (0.805 – 0.822) | 0.765  (0.748 – 0.781) | 0.863  (0.854 – 0.871) | 0.849  (0.841 – 0.857) | 0.785  (0.773 – 0.797) |
|  | SP-CNN(Ext) | 0.885  (0.877 – 0.892) | 0.813  (0.803 – 0.822) | 0.779  (0.759 – 0.799) | 0.846  (0.835 – 0.858) | 0.837  (0.828 – 0.847) | 0.793  (0.779 – 0.807) |
| Test C | **MP-CNN** | **0.772**  **(0.759 – 0.786)** | 0.663  (0.648 – 0.678) | 0.937  (0.923 – 0.950) | 0.389  (0.352 – 0.426) | 0.607  (0.594 – 0.620) | 0.864  (0.845 – 0.883) |
|  | SP-CNN(Neu) | 0.765  (0.747 – 0.782) | 0.642  (0.623 – 0.661) | 0.942  (0.926 – 0.957) | 0.343  (0.301 – 0.384) | 0.591  (0.576 – 0.605) | 0.861  (0.827 – 0.895) |
|  | SP-CNN(Flx) | 0.746  (0.729 – 0.764) | 0.643  (0.630 – 0.655) | 0.917  (0.901 – 0.934) | 0.370  (0.337 – 0.402) | 0.592  (0.583 – 0.602) | 0.7823  (0.798 – 0.848) |
|  | SP-CNN(Ext) | 0.722  (0.707 – 0.737) | 0.655  (0.641 – 0.669) | 0.884  (0.863 – 0.904) | 0.426  (0.391 – 0.461) | 0.608  (0.596 – 0.619) | 0.790  (0.767 – 0.813) |

Abbreviations: AUROC, area under the receiver operating characteristics curve; PPV, positive predictive value; NPV, negative predictive value; CI: confidence interval; MP-CNN, multi pose-based convolutional neural network model; SP-CNN, single pose-based convolutional neural network; Neu, neutral posture; Flx, flexion posture; Ext, extension posture.)
